# Long-term clinical outcome of 103 patients with acromegaly after pituitary surgery

**DOI:** 10.1007/s11102-025-01503-6

**Published:** 2025-02-22

**Authors:** Anna Pennlund, Daniela Esposito, Thomas Olsson Bontell, Thomas Skoglund, Tobias Hallén, Helena Carén, Gudmundur Johannsson, Daniel S. Olsson

**Affiliations:** 1 Department of Clinical Pathology, Sahlgrenska University Hospital, Gula stråket 8, Gothenburg, 413 45 Sweden; 2 Department of Internal Medicine and Clinical Nutrition, Sahlgrenska Academy, University of Gothenburg, Gothenburg, Sweden; 3 Department of Endocrinology, Sahlgrenska University Hospital, Gothenburg, Sweden; 4 Department of Physiology, Institute of Neuroscience and Physiology, Sahlgrenska Academy, University of Gothenburg, Gothenburg, Sweden; 5 Department of Neurosurgery, Sahlgrenska University Hospital, Gothenburg, Sweden; 6 Department of Clinical Neuroscience, Institute of Neuroscience and Physiology, University of Gothenburg, Sahlgrenska Academy, Gothenburg, Sweden; 7 Sahlgrenska Center for Cancer Research, Department of Medical Biochemistry and Cell Biology, Institute of Biomedicine, Sahlgrenska Academy, University of Gothenburg, Gothenburg, Sweden

**Keywords:** Acromegaly, Growth hormone, Pituitary adenoma, Pituitary neuroendocrine tumor, Transsphenoidal surgery

## Abstract

**Purpose:**

Acromegaly is a rare disease that can be challenging to treat due to residual pituitary adenoma after surgery or variable response to medical treatments. The primary aim of the study was to evaluate the path of treatment and long-term outcome of acromegaly after pituitary surgery.

**Methods:**

Patients with acromegaly who had undergone surgery for a growth hormone-producing pituitary neuroendocrine tumor also known as a pituitary adenoma, at Sahlgrenska University Hospital between 1994 and 2019 were included in the study. Medical records from diagnosis to the end of study (November 2022) were reviewed for surgical outcome and treatment patterns related to acromegaly.

**Results:**

In the cohort of 103 patients, 111 surgeries were performed. Mean follow-up duration was 12.7 (range: 0–37) years. Lesions were identified as a macroadenoma in 76 (76.8%) cases. At post-surgical follow-up until discharge from hospital, surgical complications and new pituitary hormone deficiency or syndrome of inappropriate antidiuretic hormone secretion occurred in 37% of cases. At 1-year post-surgery follow-up, 50% of evaluable patients achieved biochemical control of acromegaly. Of the 96 patients who had follow-up > 1 year, 53 (51.5%) had no additional medication for acromegaly after surgery until end of their follow-up. From diagnosis to the end of follow-up, 53 patients received medical therapy and seven were treated with radiotherapy.

**Conclusion:**

About half of the patients had biochemical control of acromegaly 1-year post-surgery. Treatment patterns reflected the complexity of post-surgical management and provided an overview of the long-term clinical progression in patients with acromegaly following pituitary surgery.

**Supplementary Information:**

The online version contains supplementary material available at 10.1007/s11102-025-01503-6.

## Introduction

Acromegaly is most often caused by a growth hormone (GH)-producing pituitary neuroendocrine tumor (pit-NET) also known as a pituitary adenoma. The incidence of acromegaly varies between 0.2 and 1.1/100 000 individuals/year [1, 2]. Surgical resection of the adenoma may result in disease control but not in all cases [3–5], especially for macroadenomas where remission rates are reported between 45 and 70% [5]. Long-term uncontrolled GH-production can result in excess mortality and comorbidities such as increased risk of cardiovascular disease and diabetes [6–10]. Pit-NETs may co-produce GH (also called somatotropin or somatotropic hormone) and prolactin, and they express the pituitary-specific positive transcription factor 1 (PIT1) [11, 12].

Second-line treatment after pituitary surgery is usually medical treatment. This includes somatostatin receptor ligands (SRLs) [13] and sometimes combination treatments of GH-receptor antagonists, dopamine agonist or treatment with second-generation SRL pasireotide [14] in some patients to obtain biochemical control. All treatment regimens can have side effects, for example hypopituitarism after pituitary surgery [15] or radiation therapy [16, 17].

Known predictive factors of poor outcome include local tumor invasion, subtype sparsely granulated somatotroph tumor, and plurihormonal tumors of *PIT1* lineage [11]. Previous studies have compared the pituitary surgical techniques, microscopic transsphenoidal surgery and endoscopic transsphenoidal surgery: showing differences in complication pattern [18] however, some with no difference in outcome for remission rates [19, 20]. In a recent study [21], the long-term rate of biochemical control of acromegaly with surgery alone was 37–58%.

The primary aim of our study was to determine the long-term remission rate, treatment pattern, and complications in a cohort of patients who underwent surgery for GH- or GH/prolactin-secreting pit-NETs at our hospital. A secondary aim was to identify subgroups of patients with acromegaly with aggressive illness to allow for comparison of clinical and molecular characteristics between the groups in future work.

## Materials and methods

This retrospective study included patients who had undergone surgery for a GH- or GH/prolactin-secreting pit-NET at Sahlgrenska University Hospital (Gothenburg, Sweden) during the period 1994–2019. Medical records for the defined cohort spanned 1984–2022. Exclusion criteria were: age < 18 years when diagnosed with the pit-NET; use of neoadjuvant radiotherapy for the pituitary adenoma before surgery; adenoma with a diagnosis of mixed hormone-production that was not GH/prolactin; mixed ganglioglioma and pit-NET; no histological verification of a pit-NET in tissue from the surgery during initial screening; and surgery before computerization of medical records by the Pathology Department in 1994.

### Data collection

The cohort was identified from medical records retrieved in lists originating from three separate digital record systems used by the Endocrinology, Neurosurgery, and Pathology Departments, respectively. Pre-operative radiological statements and laboratory tests were also collected.

Information concerning which assays used for analyzing insulin-like growth factor-1 (IGF-1) during the study period were collected from the Department of Clinical Chemistry of Sahlgrenska University Hospital. The successive assays used to assess IGF-1 during the study period were Nichols RIA (1994–2003), Nichols Advantage (2003–2006), Siemens Immulite 2500 (2006–2011), Siemens Immulite 2000XPi (2011–2013) and IDS-iSYS IGF1 IS-3900 (2013–2022). The successive GH assays used during the study period were hGH RIA kit 10-6409-01 (1994–1995), DELFIA hGH kit nr 1244.041 (1995–2008), and Reagent Access GH, ref 33,580 (2008–2022).

Follow-up data for the patients was collected from clinical records (computer and physical archives) concerning: (1) the overall follow-up period; (2) data from laboratory and radiological examinations; and (3) follow-up data connected to acromegaly diagnosis and treatment. Data was extracted for standardized follow-up times: 3 months, and 1, 2, 5, and 10 years from the time of surgery. Due to the long study period and multiple factors causing displacement of follow-up schedules, the set deviation of time frame was for 3 months (1–7 months post-surgery), and 1 year (± 3 months), 2 years (± 6 months), 5 years (± 9 months), and 10 years (± 12 months).

### Data analysis

To overcome assay-, age- and sex-dependent differences in numerical reference values, a patient’s IGF-1 level was defined as a quotient of the upper limit of normal (ULN) for the age- and sex-adjusted IGF-1 level. Since the study period spans approximately three decades, the criteria for diagnosis and control of disease has varied across this time [22–24]. In our study, the criteria for defining biochemical control of acromegaly is an IGF-1 value within the age- and sex-adjusted reference or GH < 0.4 µg/L during an oral glucose tolerance test (OGTT) based on guidelines from 2010 [23]. Levels of IGF-1 > 1.3 × ULN were also identified due to the most recent 2023 consensus criteria [25, 26], which classifies this level and features typical for acromegaly as criteria for the disease. Due to a unit change of the GH-assays, where the test from 1993 to 2008 used mU/L instead of µg/L, a conversion was needed. The calculation used for conversion between the units was: GH in µg/L × 3 = GH in mU/L, as based on a consensus from year 2006 [27]. Assay variability was not taken into account for the IGF-1 and GH assays.

Adenomas were classified as invasive or not depending on the clinical records containing radiologists’ statements. The lesion was considered invasive if it was linked to: (1) descriptions such as “invasive”, “ingrowth in sinus”, “surrounding the carotid artery”; (2) use of Knosp classification [28], reaching grade 3 or 4 of cavernous sinus invasion; or (3) described as SIPAP classification [29] 3 or 4 in the parasellar space or 2 in the infrasellar space.

Histopathological diagnoses of the cases reflect the classification used at the time of diagnosis. Therefore, the adenomas were grouped only as: GH-producing or with co-production of prolactin based on immunohistochemical (IHC) expression of the proteins.

## Results

### Study population

Initial screening of the retrieved patient lists resulted in 262 patients who were eligible for further in-depth assessment. A total of 159 patients were excluded: the causes for exclusion are available in Supplementary Material 1. A total of 103 patients (60 men, 43 women) met the inclusion criteria and had a mean age of 45.7 (range: 23–75) years. The mean age of the women was 44 (range: 23–71) years and the men 47 (range: 24–75). The age of one patient at diagnosis was not identifiable, since the patient had presented with symptoms outside of Sweden and did not remember the date of diagnosis, nor were original copies of medical records available.

**Fig. 1:**
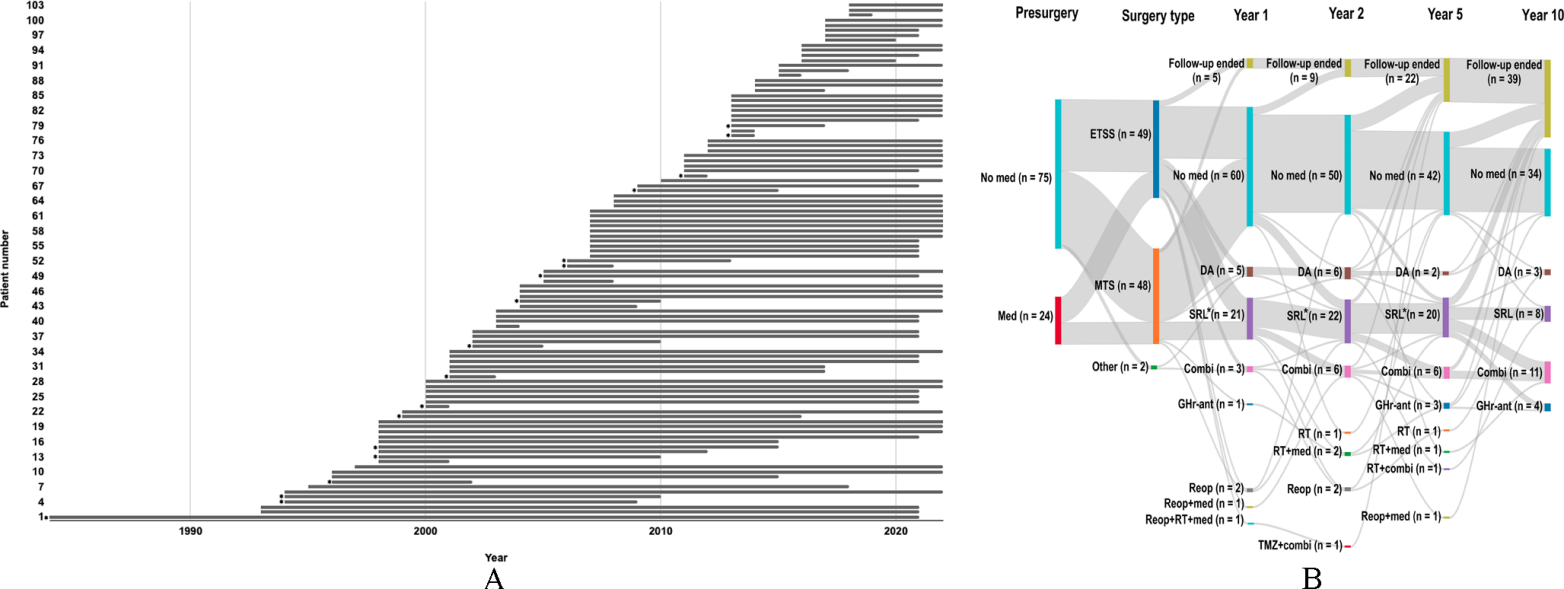
Overview of the long-term follow-up. **(A)** Timeline (1984–2022) of patient follow-up from initial diagnosis to last follow-up. Two patients had their initial diagnosis in another country but, in the chart, this is depicted from the time their diagnosis was established in Swedish medical care. Patients may still be under surveillance in 2022 even though their latest follow-up was in, for example, 2021. *Patient deceased at the end of the study period. **(B)** Treatment pattern on a group level for the cohort. Nodes represent: (1) medication or not before first surgery at Sahlgrenska University Hospital; (2) surgery type; and node 3–6) current treatment option at follow-up 1-, 2-, 5- and 10-year post-surgical follow-up. Abbreviations: Combi, combination therapy (SRL and/or DA and/or pegvisomant); DA, dopamine agonist; ETSS, endoscopic transsphenoidal surgery; GHr-ant, GH-receptor-antagonist/pegvisomant; Med, medication for acromegaly; MTS, microscopic transsphenoidal surgery; Reop, reoperation; SRL, somatostatin receptor ligand; RT, radiotherapy; TMZ, temozolomide. *, Including treatment with second generation somatostatin receptor ligand

The duration of follow-up for individuals in the cohort is presented in Fig. 1A. All surgical treatments were primary treatments except for two individuals who had already undergone previous surgery at other centers. A total of 93 patients (90.3%) underwent a single surgery and the remaining underwent two (*n* = 9) or three (*n* = 1) surgeries. All of the cases of acromegaly were sporadic, except one patient who was diagnosed with multiple endocrine neoplasia type 1.

### Radiological characteristics

Pre-operative magnetic resonance imaging was available for adenoma size measurement in 99 of 103 patients: a macroadenoma was identified in 76 (76.8%) and microadenoma in 23 (23.2%). It was possible to interpret invasiveness in 97 of 103 patients: the adenoma was locally invasive in 46 (47.4%) and non-invasive in 51 (52.6%).

**Table 1:** Adenoma characteristics and patient gender by type of IHC hormone expression of 101 cases

Parameter	STH (*n* = 53)	STH/PRL (*n* = 46)	PRL* (*n* = 2)
Mean (range) IGF-1, × ULN	3.20 (1.10–6.12)^†^	2.81 (1.03–5.88)^‡^	2.53 (2.40–2.66)
Size on MRI, *n* (%)			
Macroadenoma	43 (82.7)	31 (70.5)	2 (100)
Microadenoma	9 (17.3)	13 (29.5)	0
Data missing	1	2	0
Invasive, *n* (%)			
Yes	24 (48.0)	20 (45.5)	2 (100)
No	26 (52.0)	24 (54.5)	0
Data missing	3	2	0
Sex, *n* (%)			
Male	30 (56.6)	28 (60.9)	1 (50.0)
Female	23 (43.4)	18 (39.1)	1 (50.0)

*Patients only had positive stain for prolactin but had verified GH overproduction. IGF-1 ULN value for these patients: Patient 1: 2.7. Patient 2: 2.4

Missing data: ^†^*n* = 5; ^‡^*n* = 3

Abbreviations: GH, growth hormone; IGF-1, insulin-like growth factor-1; IHC, immunohistochemistry; PRL, prolactin; STH, somatotropin: ULN, upper limit of normal

### Histological diagnoses

The majority of interventions (96/111; 86.5%) were performed before 2016. IHC stains for transcription factors were therefore not available for most cases and only a low frequency stained for cytokeratin CAM5.2. Most of the histological diagnoses in the cohort were based on hematoxylin and eosin, and IHC stains for somatotropin, prolactin, and adrenocorticotropic hormone. Some cases were also stained with antibodies against thyroid-stimulating hormone. The following groups were therefore constructed: somatotropin, somatotropin/prolactin, and prolactin. Distribution of cases is presented in Table 1. Of the 103 patients, 101 had a histopathological hormone profile for diagnosis. For the two patients who did not, one of which was simply described as “relapse of pituitary adenoma” and, in the other, the tissue was not definitely identified as adenoma but was described as a macroadenoma on magnetic resonance imaging.

### Surgical outcome

For the cohort of 103 patients, 111 surgeries were performed during 1994*–*2022 at Sahlgrenska University Hospital. Three additional interventions were performed in other centers. Ten patients underwent reoperation during their course of disease. All but two of the 111 surgeries were performed by neurosurgeons or a cooperation between neurosurgeons and otorhinolaryngologists. Two of the operations were performed by otorhinolaryngologists alone with the purpose of obtaining a biopsy specimen. In the cohort, 101 patients had their first pituitary surgery at Sahlgrenska University Hospital within the study period. A total of 28 individuals had medical treatment for acromegaly before any of their surgeries although only 24 of these had sufficiently complete records to be included in Fig. 1B. The dominant surgical technique during the period shifted away from microscopic transsphenoidal surgery to endoscopic transsphenoidal surgery. Mean hospital stay in the neurosurgical and endocrine ward all together was 12 (range: 4–30) days.

**Table 2:** Post-surgical complications until discharge from hospital

	No. (%)
Total patients evaluated*	100
Complication	
Surgical	15 (15)
Hormonal	25 (25)
Surgical and/or hormonal	37 (37)
Type of surgical complication	
Arrhythmia	1 (1)
Epistaxis^†^	4 (4)
Liquorrhea	6 (6)
Local infection	2 (2)
Meningitis	1 (1)
Pulmonary embolism	1 (1)
Paresthesia	2 (2)
Stroke	1 (1)
Type of hormonal complication	
Cortisol substitution	3 (3)
Diabetes insipidus	8 (8)
Gonadotropin deficiency	4 (4)
SIADH	12 (12)
Thyrotropin substitution	2 (2)

Note: some individual patients suffered more than one surgical/hormonal complication. *Missing data (*n* = 3). ^†^Epistaxis severe enough to require medical care. Abbreviation: SIADH, syndrome of inappropriate antidiuretic hormone secretion

Post-surgical complications (surgical and/or hormonal) until discharge from hospital are summarized in Table 2. For the primary surgeries, only one required premature termination due to excessive bleeding at the surgical site. Surgical complications occurred for 15% of procedures. Six patients had suspected liquorrhea but only two of them required treatment (one with lumbar drain alone and the other with lumbar drain and reoperation). Hormonal disruptions, including either an excess or deficiency of anti-diuretic hormone and hypopituitarism necessitating hormone replacement therapy, were observed in 25% of cases. Of the eight cases of diabetes insipidus, five patients were still treated with desmopressin around the time of their 3-month post-surgical follow-up. Counting cases with surgical and/or hormonal complications together, the rate of complications was 37%.

**Table 3:** Number of patients with records at follow-up times and reasons for missing records

	Follow-up time				
	3 mo	1 yr	2 yr	5 yr	10 yr
Follow-up recorded, *n* (%)	87 (84.5)	86 (83.5)	80 (77.7)	69 (67.0)	59 (57.3)
Reason for no follow-up, *n* (%)					
Out of time frame	11 (10.7)	8 (7.8)	8 (7.8)	5 (4.9)	1 (1.0)
Deceased	1 (1.0)	1 (1.0)	2 (1.9)	4 (3.9)	6 (5.8)
Referral/discontinuation	3 (2.9)	6 (5.8)	8 (7.8)	11 (10.7)	13 (12.6)
Disappeared from follow-up	0	1 (1.0)	2 (1.9)	4 (3.9)	5 (4.9)
Record not found	1 (1.0)	1 (1.0)	3 (2.9)	4 (3.9)	0
Not enough time passed	0	0	0	6 (5.8)	19 (18.4)

Only displaying deaths within the stated time periods

Abbreviations: mo, month; yr, year

**Table 4:** Hormone replacement therapies at follow-up

	Follow-up time			
	1 yr (*n* = 86)	2 yr (*n* = 80)	5 yr (*n* = 69)	10 yr (*n* = 59)
Hormone substitution, *n* (%)				
Growth hormone	0	3 (3.8)	1 (1.4)	2 (3.4)
Thyroxine	13 (15.1)	14 (17.5)	14 (20.3)	19 (32.2)
Cortisone	4 (4.7)	6 (7.5)	5 (7.2)	5 (8.5)
Sex hormone	18 (20.9)	18 (22.5)	21 (30.4)	18 (30.5)
Desmopressin	1 (1.2)	1 (1.3)	0	0

Abbreviation: yr, year

There were 88 patients who had a recorded hospital contact between the time of discharge up to 3 months post-surgery. One patient had a complication 1 week after discharge but no visit within the 3-month follow-up interval, which accounts for the discrepancy in Table 3. In this cohort, four (4.5%) individuals suffered epistaxis sufficiently severe to the point medical assistance was needed and four (4.5%) other individuals contracted a local infection in relation to the operated area. One (1.1%) additional patient had bleeding in the residual adenoma and also had an ischemic stroke. No mortalities related to surgery were identified during this period. Apart from the aforementioned complications, 13 out of the total 88 patients (14.8%) complained about subjective symptoms but did not experience any severe complications. The most common complaint noted in the medical records was headache or pain (*n* = 7). Other subjective symptoms were tiredness and nasal congestion.

### Long-term follow-up

In November 2022, 68 patients (66%) of the initial cohort were still under active monitoring. The average follow-up duration was 12.7 (range: 0–37) years. The cause of no recorded follow-up at the set follow-up times is presented in Table 3. The most frequent cause of no recorded follow-up at 10 years post-surgery was that insufficient time had passed since the intervention.

A total of 53 (51.5%) patients who had a follow-up time of ≥ 1 year did not receive any further medical treatment post-surgery, whereas 43 (41.7%) were given additional treatment for acromegaly during their specific follow-up period. Four cases (3.9%) could not be evaluated due to follow-up < 1 year and three cases (2.9%) were not evaluated due to missing records or did not receive surgery by a neurosurgeon. In this subgroup of patients without further medication, 14 cases were identified as microadenomas and 38 as macroadenomas, and one case did not have data on adenoma size.

For the evaluation of biochemical control of the disease, 74 (71.8%) patients reached biochemical control of acromegaly (IGF-1 ≤ ULN) with multimodal treatment during their follow-up, whereas 15 patients (14.6%) did not. Overall, parts of the medical records were not obtained for 11 (10.7%) cases; thus, no certain conclusion could be made concerning biochemical control. For three (2.9%) patients no post-surgical follow-up was recorded. At 1-year post-surgery, 86 patients had a recorded follow-up and 43 (50.0% of that group but 41.7% of the total cohort) patients had obtained biochemical control of acromegaly. For those patients with data on invasiveness and biochemical remission at one year follow-up, 40% (16 out of 40 patients) with invasive tumors and 71.4% (30 out of 42 patients) of those without invasive tumors reached biochemical control at one year follow-up. At the 2-year post-surgery follow-up, there were 78 records where laboratory results were found and two cases where the laboratory results were not located, which accounts for 80 patients followed up in Table 3: 49 (62.8% of that group but 47.6% of the total cohort) were under biochemical control. At 5-years post-surgery follow-up, 69 patients had a recorded appointment and 45 (65.2% of that group but 43.7% of the total cohort) patients had biochemical control, although 11 (10.7% of the total cohort) patients still had an IGF-1 > 1.3× ULN at this follow-up. In these 11 uncontrolled patients, the mean IGF-1 ULN was 2.0 (range 1.3–3.6). Out of these uncontrolled patients, 7 had either medical treatment or had prior received radiation therapy. The information about if the remaining 4 patients had intolerance, adherence issues or specific patient preferences is missing, but their IGF-1 ULN were all below 1.8.

### Treatment pattern

The active treatments given to the cohort at set times during the follow up (1, 2, 5 and 10 years post-surgery) are displayed at a group level in Fig. 1B. A total of 99 (96.1%) patients had records complete enough to be included in the figure. One individual with a GH- and prolactin-secreting pit-NET required chemotherapy with temozolomide during their recorded follow-up. A total of 53 individuals received some type of medical treatment for acromegaly between the time of diagnosis to the end of their follow-up. The most commonly administered medical treatment to the 103 patients was an SRL. Specific treatments during follow-up were SRLs (*n* = 50, 48.5%, out of which two patients received 2nd generation SRL), combination therapy (*n* = 23, 22.3%), dopamine agonists (*n* = 21, 20.4%), radiotherapy (*n* = 7, 6.8%) and temozolomide (*n* = 1, 1.0%). Multiple different types of combination medical therapy were used, the two most common combinations were 1st Generation SRL combined with GH receptor antagonist (*n* = 16) and 1st generation SRL combined with dopamine agonist (*n* = 7). Radiotherapy was given to 7 patients, of which three received traditional fractionated radiotherapy, two received treatment with gammaknife and two received stereotactic single fraction radiotherapy.

During the study period, hormone substitutions were given according to the numbers in Table 4. The most frequent substitution at all times of follow-up were sex hormones. At 1-year follow-up, only one patient needed desmopressin and no patients had GH substitution. Cortisol substitution was required by four to six patients at different follow-up times.

During long-term follow-up, two patients had suspected cerebral infections. One of them developed a cerebral fungal infection, which caused a mycotic aneurysm leading to a fatal brain bleed. The other had suspected cerebral abscesses where the condition worsened, then stabilized, and then the patient died suddenly (no infectious agent was identified). Both events occurred 15 years after pituitary surgery and could not be clearly linked to the intervention.

## Discussion

The main finding of this study is that 71.8% of patients obtained biochemical control of acromegaly with multimodal treatment during their follow-up and the majority of individuals remained in the surveillance program. The percentage of patients in our cohort (60.6%; Fig. 1B) who had no medical treatment 1-year post-surgery is in line with numbers reported from a recent Norwegian study concerning rates of biochemical control [21]. It should be noted that “no active treatment” does not always equal IGF-1 and GH within the reference criteria for biochemical control. In our cohort, at 1-year post-surgery, 50% of evaluable patients met the criteria for biochemical control of acromegaly. This result is partially based on the criteria of GH of < 0.4 µg/L on oral glucose tolerance test. Consequently, it is possible the percentage of patients with biochemical control would have been higher if the GH cut-off at < 1.0 µg/L was used, which previously applied in our clinic. Response rate differs somewhat with respect to 5-year post-surgical follow-up compared to the Norwegian study [21], where our cohort showed that 42.4% of patients did not have additional treatment compared to 52.5%. We note that our cohort has a number of patients lost to follow-up, i.e., at 5 years post-surgery: at that time, the follow-up of 11 (10.7%) patients had been discontinued or they had transferred to another hospital (Table 3). As pituitary surgery is regionalized in our country, it can be speculated that some patients may have had to travel far and wished to have follow-up closer to home– it may be that patients with more manageable disease are the ones who follow this route.

A substantial portion of patients remained with residual tumor activity after primary intervention, although relatively few patients required reoperation (*n* = 10, 9.7%) or radiotherapy (*n* = 7, 6.8%). The subgroup of 11 patients with acromegaly who still had IGF-1 ≥ 1.3 ULN at 5 years post-surgery would, based on the consensus guidelines from 2023 [25, 26], still fulfil criteria for disease if symptoms were present. Similar results were reported in a French study of patients with acromegaly by Albarel and colleagues [30], where nine out of 115 (7.8%) patients had uncontrolled disease in their long-term follow-up.

The rates of peri- and post-operative complications after intervention, such as diabetes insipidus were similar to previous reports [31, 32]. Liquorrhea, in our study, was based on clinical assessment at the time, since the testing for beta-trace protein was not available during the majority of the time period. With respect to the two cases of cerebral infections, autopsies were performed. In the individual diagnosed with fungal infection, autopsy revealed that the sphenoid bone was indeed engaged in the infection. The second case did not show any clear signs of cerebral infection nor involvement of the sella region. It is not known if these events were a coincidence or related to the previous transsphenoidal intervention. Little support was found in the literature, albeit two articles reporting late cerebral infection with suspected relation to pituitary surgery do exist [33, 34].

Since the 1990s, laboratory and histopathological diagnostics have undergone changes. In the newest editions of WHO Classification of Tumors [12], pit-NETs are subgrouped according to transcription factor expression, which was not the case for all previous editions during the study period. In our cohort, no staining for transcription factors was performed and cytokeratin granulation patterns were only available for about 10% of cases. This limits further investigation of subgroups in this study.

It should be kept in mind when reading this paper that it represents a selected cohort in a limited geographical location. The inclusion criteria of “surgical intervention”, will bias towards cases that had a baseline possibility of cure by surgery, needed surgical decompression, or required surgery due to side effects of medications or resistance to medical treatment. With respect to treatment patterns and outcome, the impact of the long timeframe must be taken into consideration. The period of study spans eras with different guidelines and assays as well as changes in the treatment arsenal. Treatment with second-generation SRLs [35] and GH-receptor antagonists [36, 37] were put into practice during the study period, and radiotherapy usage has changed [38, 39]. Recent articles suggest that mortality for the patient group has decreased during the latter part of our study period [9, 40, 41]. Despite these limitations, the study consists of a large cohort of patients with acromegaly, of whom a substantial proportion have many years of follow-up and their diagnosis is verified with laboratory results and tissue specimens.

## Conclusions

In the cohort of over 103 patients with acromegaly who were eligible for pituitary surgery from 1994 to 2019 at Sahlgrenska University Hospital, about half of the individuals were under biochemical control 1-year post-surgery. In this real-life cohort, 53 of 103 patients required some sort of medical treatment for acromegaly during the course of their illness, with some requiring combination therapy. The overall follow-up rate of patients with surgically treated acromegaly was high, with only a few individuals lost to follow-up. The cohort contains a small proportion of patients with uncontrolled acromegaly despite 5 years of active follow-up and only a handful of cases required radiation therapy. The study clearly demonstrates the complexity of treatment for many patients with acromegaly, which includes both medications targeted at obtaining disease control but also treatment of hypopituitarism. The study may provide a basis for further analysis of molecular patterns of the resected tissue and correlate outcome to clinical variables such as such as disease resistant to medical treatments.

## Electronic supplementary material

Below is the link to the electronic supplementary material.

Supplementary Material 1

## Data Availability

No datasets were generated or analysed during the current study.
