# Supplementary material for: Long-term clinical outcome of 103 patients with acromegaly after pituitary surgery
Source: Pituitary. 2025 Feb 22;28(2):33. doi: 10.1007/s11102-025-01503-6 (PMC11846723; doi:10.1007/s11102-025-01503-6)

Long-term clinical outcome of 103 patients with acromegaly after pituitary surgery

Authors: Anna Pennlund[1,2,*]**,** Daniela Esposito[2,3]**,** Thomas Olsson Bontell[1,4]**,** Thomas Skoglund[5,6]**,** Tobias Hallén[5,6]**,** Helena Carén[7]**,** Gudmundur Johannsson[2,3]**,** and Daniel S. Olsson[2,3]

Affiliations:
[1]Department of Clinical Pathology, Sahlgrenska University Hospital, Gothenburg, Sweden
[2]Department of Internal Medicine and Clinical Nutrition, Sahlgrenska Academy, University of Gothenburg, Gothenburg, Sweden
[3]Department of Endocrinology, Sahlgrenska University Hospital, Gothenburg, Sweden
[4]Department of Physiology, Institute of Neuroscience and Physiology, Sahlgrenska Academy, University of Gothenburg, Gothenburg, Sweden
[5]Department of Neurosurgery, Sahlgrenska University Hospital, Gothenburg, Sweden
[6]Department of Clinical Neuroscience, Institute of Neuroscience and Physiology, University of Gothenburg, Sahlgrenska Academy, Gothenburg, Sweden
[7]Sahlgrenska Center for Cancer Research, Department of Medical Biochemistry and Cell Biology, Institute of Biomedicine, Sahlgrenska Academy, University of Gothenburg, Gothenburg, Sweden

***Correspondence to:** Anna Pennlund, Department of Clinical Pathology, Sahlgrenska University Hospital, Gula stråket 8, 413 45 Gothenburg, Sweden. Email: anna.pennlund@gu.se ORCiD: 0000-0002-9700-2381

Supplementary material 1. Flowchart of the patient selection process. Abbreviations: ACTH, adrenocorticotropic hormone; GH, growth hormone; Pit-NET, pituitary neuroendocrine tumor; TSH, thyroid-stimulating hormone.

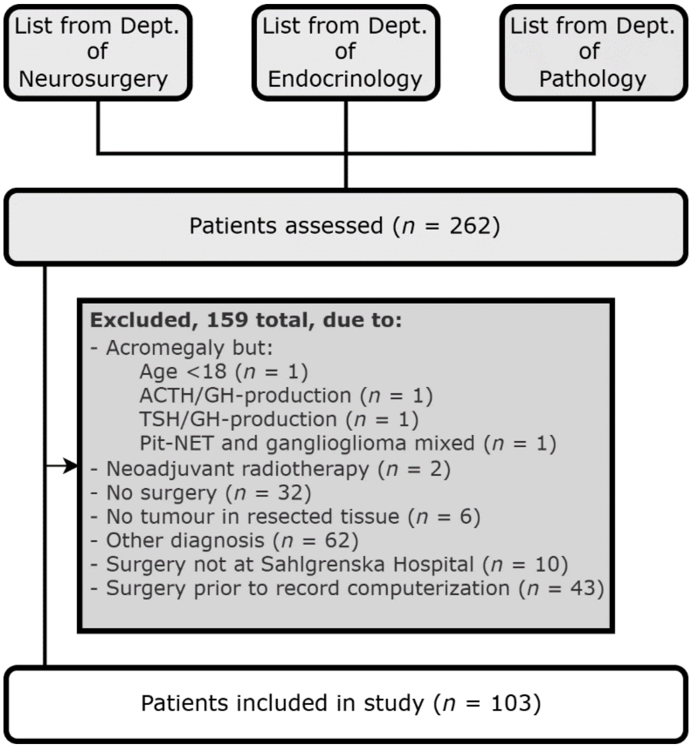

Supplement: Supplementary file 1 — Supplementary Material 1 [file 11102_2025_1503_MOESM1_ESM.pdf]
